# The Emerging Role of Magnesium in Preventing Acute Kidney Disease During Concurrent Chemoradiotherapy in Head and Neck Cancer

**DOI:** 10.3390/cancers17203310

**Published:** 2025-10-14

**Authors:** Francesco Trevisani, Andrea Angioi, Matteo Floris, Sara Cardellini, Leone Giordano, Alberta Culiersi, Agnese Monti, Aurora Mirabile

**Affiliations:** 1 Department of Urology, Division of Experimental Oncology, IRCCS San Raffaele Scientific Institute, 20132 Milan, Italy; 2 Urological Research Institute URI, IRCCS San Raffaele Scientific Institute, 20132 Milan, Italy; agnesemonti02@gmail.com; 3 Department of Nephrology, Dialysis, and Transplantation, ARNAS G. Brotzu, 09047 Cagliari, Italy; andrea.angioi@aob.it (A.A.); matteo.floris@aob.it (F.M.); 4 Clinical Nutrition, IRCCS San Raffaele Scientific Institute, 20132 Milan, Italy; cardellini.sara@hsr.it; 5 Department of Otorhinolaryngology, IRCCS San Raffaele Scientific Institute, 20132 Milan, Italy; giordano.leone@hsr.it (G.L.); culiersi.alberta@hsr.it (C.A.); mirabile.aurora@hsr.it (M.A.)

**Keywords:** AKD, renal function, head and neck cancer, onconephrology, chemotherapy

## Abstract

**Simple Summary:**

Cisplatin is widely used in the treatment of head and neck cancer, but its use is often limited by kidney toxicity. Acute kidney disease is a condition that can appear after cisplatin exposure and may compromise treatment completion and long-term outcomes. In this study, we prospectively followed patients receiving high-dose cisplatin with radiotherapy under a standardized supportive care protocol that included intravenous magnesium. We observed that some patients still developed kidney problems, and that lower baseline kidney function, higher body weight, and the exposure to selected medications were linked to a greater risk. Magnesium supplementation was well tolerated and was associated with reduced kidney injury. These results underline the importance of careful monitoring and provide evidence that magnesium could be a simple and accessible strategy to improve the renal safety of cisplatin-based therapy in head and neck cancer.

**Abstract:**

Background: High-dose cisplatin (≥200 mg/m^2^ cumulative) remains the standard of care in concurrent chemoradiotherapy (CRT) for locally advanced head and neck squamous cell carcinoma (LA-HNSCC). However, its use is frequently limited by nephrotoxicity, including acute kidney disease (AKD). This recently described clinical renal syndrome encompasses functional alterations of the kidney lasting fewer than 3 months post-exposure. Although hydration protocols and antiemetic strategies are routinely applied to avoid reduction in oral liquid intake and to prevent dehydration that could worsen renal function, AKD continues to pose a threat to reach the therapeutic dose, to treatment completion, and long-term outcomes. Recent evidence supports the nephroprotective role of intravenous (IV) magnesium in mitigating cisplatin-induced tubular injury, yet prospective data on its impact in real-world LA-HNSCC settings remain limited. We aimed to prospectively investigate the incidence and characteristics of renal impairment, particularly AKD, in a real-world cohort of LA-HNSCC patients treated with high-dose cisplatin and standardized supportive therapy, including intravenous magnesium. Methods: We conducted a prospective observational study including 207 patients with LA- HNSCC undergoing high-dose cisplatin-based CRT (≥200 mg/m^2^ cumulative dose), within a standardized supportive care protocol incorporating IV magnesium. Renal function was assessed over three cycles via serum creatinine and estimated glomerular filtration rate (eGFR). AKD was defined and staged according to KDIGO criteria. Clinical and biochemical predictors of AKD were explored. Results: AKD occurred in 5.3% of patients (11/207; 95% CI 2.7–9.3), with eight events between C1→C2, 3 between C2→C3, and 0 thereafter; recovery at the next cycle was 9.1% (1/11). Among them, 57.1% were classified as stage 1. A baseline eGFR < 90 mL/min/1.73 m^2^ was associated with a higher AKD incidence (13.3% vs. 5.4%). Body mass index (BMI) was significantly associated with AKD in univariate analysis (*p* = 0.02), whereas no independent predictor emerged in multivariate analysis. Use of renin–angiotensin–aldosterone system (RAAS) inhibitors was more frequent among patients who developed AKD (*p* = 0.04). Renal function declined more steeply in AKD patients, with a median eGFR slope of −0.3917 mL/min/1.73 m^2^/day vs. −0.0483 mL/min/1.73 m^2^/day in those without AKD (*p* = 0.0005), irrespective of CKD stage. Conclusions: In a real-world cohort receiving high-dose cisplatin with structured nephroprotection including IV magnesium, AKD developed in approximately 10% of patients. Lower baseline eGFR, elevated BMI, and RAAS inhibitor use emerged as potential risk factors. These findings reinforce the importance of proactive renal monitoring and suggest a role for magnesium supplementation as an accessible strategy to enhance renal safety in curative-intent CRT.

## 1. Introduction

Concurrent chemoradiotherapy (CRT) with high-dose cisplatin remains the standard therapeutic approach for patients with locally advanced head and neck squamous cell carcinoma (LA-HNSCC), offering curative potential across both HPV (Human Papillomavirus)-positive and HPV-negative disease subsets. The tri-weekly regimen (100 mg/m^2^ every 21 days for three cycles) has demonstrated superior oncologic efficacy in terms of locoregional control and overall survival when compared to weekly low-dose strategies or alternative agents, especially in an up-front approach [1,2,3]. However, the clinical utility of cisplatin is frequently challenged by its narrow therapeutic index and significant toxicity profile, foremost among which is nephrotoxicity [4,5].

Patients with LA-HNC are further predisposed to complications due to the synergistic impact of CRT-related toxicity on nutritional intake. Severe oral mucositis, nausea, vomiting, diarrhea and odynophagia lead to a reduction in oral liquids intake and to dehydration that could worsen renal function.

Cisplatin-induced acute kidney injury (AKI) and acute kidney disease (AKD) are increasingly recognized complications with substantial implications for oncologic outcomes. While AKI refers to an abrupt decline in glomerular filtration within 7 days, AKD encompasses persistent renal dysfunction up to 90 days after exposure and may progress to chronic kidney disease (CKD) if unresolved [6,7]. The nephrotoxic effects of cisplatin are primarily mediated by accumulation in renal proximal tubular epithelial cells, where it induces mitochondrial damage, oxidative stress, DNA damage, and activation of proinflammatory and profibrotic signaling cascades [8,9]. Clinical manifestations may range from subclinical reductions in estimated glomerular filtration rate (eGFR) to symptomatic uremia requiring hospitalization or discontinuation of therapy [10].

This damage can happen even with proper protective strategies and in patients who do not have baseline chronic kidney disease.

In head and neck cancer (HNC), the risk of nephrotoxicity is particularly pronounced due to the curative-intent cisplatin dosing strategies employed, necessitating a ≥200 mg/m^2^ cumulative dose [11]. Even with contemporary hydration protocols, mannitol diuresis, and antiemetic prophylaxis, real-world studies report an incidence of grade ≥ 2 AKI of 20–30%, with a non-negligible proportion developing persistent AKD [12,13]. Interruptions or delays in chemotherapy cycles due to kidney toxicity may lead to suboptimal cumulative dosing, compromising tumor control and long-term prognosis. These complications can compromise treatment completion, hinder the use of future nephrotoxic agents, and negatively impact overall survival [14].

In this challenging clinical context, recent evidence suggests a nephroprotective role for intravenous (IV) magnesium supplementation. Cisplatin induces magnesium wasting of the kidneys via downregulation of TRPM6/7 channels and damage to the distal tubules, resulting in hypomagnesemia that exacerbates kidney injury [15]. Experimental models have demonstrated that magnesium repletion preserves kidney function by stabilizing cell membranes, reducing oxidative damage, and maintaining the expression of apical efflux transporters such as MRP2 and MRP4, thereby promoting cisplatin excretion [16,17,18].

Clinical data have supported these findings. In a recent large multicenter cohort study involving 13,917 patients, Gupta et al. demonstrated that same-day administration of IV magnesium significantly reduced the incidence of moderate-to-severe AKI or death within 14 days post cisplatin infusion (adjusted OR 0.80; 95% CI, 0.66–0.97), with a greater protective effect observed in patients with preserved baseline kidney function and normal serum magnesium levels [19]. Notably, magnesium supplementation showed benefit even in patients receiving standard hydration, suggesting an additive nephroprotective mechanism.

Despite these promising insights, there is a lack of prospective data evaluating AKD incidence and kidney function progression in HNC patients treated with high-dose cisplatin within a structured nephroprotection protocol that includes magnesium. The unique demands of cisplatin-based CRT in LA-HNSCC, combined with the aggressive dosing schedule and frequent comorbidities, warrant dedicated investigation into risk mitigation strategies.

The present study aims to prospectively evaluate the incidence, timing, and clinical characteristics of AKD in a cohort of LA-HNSCC patients treated with high-dose cisplatin and radiotherapy, within a standardized hydration regimen incorporating prophylactic IV magnesium. Beyond the 7-day AKI window, cisplatin-based CRT entails repeated nephrotoxic insults across cycles. Acute kidney disease (AKD) captures this subacute continuum and has been linked to subsequent CKD and adverse outcomes in oncology cohorts [7,20,21,22]. Monitoring AKD, therefore, provides a more complete assessment of renal safety and long-term risk in curative-intent CRT. By characterizing renal outcomes in this context, we aim to contribute evidence for the systematic use of magnesium as a cost-effective and scalable intervention to enhance the renal safety of curative-intent cisplatin therapy.

## 2. Materials and Methods

A prospective study was conducted in a cohort of 207 patients enrolled at IRCCS San Raffaele Hospital in Milan between 2017 and 2025. Clinical data and laboratory tests (such as creatinine, potassium, urea, hemoglobin, leukocytes, neutrophils and lymphocytes) were collected at 3 time points (every 3 weeks, from the beginning of the treatment); BMI was calculated at the first oncological visit by dividing body weight (kg) by the height squared (m^2^) [23].

We considered the following patient data: age, gender, smoking and potus habit, hypertension, diabetes, and medical therapy (steroids, oral antidiabetic agents, insulin, proton pump inhibitors, angiotensin II receptor blockers, ASA, RAAS inhibitors, calcium antagonists, beta-blockers, and diuretics, narcotics). This study included adults aged ≥18 years with LA-HNSCC deemed medically fit for a treatment plan involving high-dose cisplatin and radiotherapy, within a standardized hydration regimen incorporating prophylactic IV magnesium. Women known to be pregnant or planning to become pregnant during the trial period were excluded from the study.

The glomerular filtration rate (GFR) was estimated at each time point (0, 1, 2) using the creatinine-based estimated glomerular filtration rate (eGFR) formula: CKD-EPI 2012 [24].

Acute Kidney Disease (AKD) was defined using the KDIGO 2024 guidelines [25].

The study received the approval of the Institutional Ethical Committee (San Raffaele Hospital, Milan, approval date 8 June 2022), and all patients included in this study signed an informed consent form. All the experimental procedures involving human biological material complied with the approved guidelines and were conducted according to good clinical practice.

### 2.1. Treatment

All patients were treated with:

- High-dose cisplatin concomitant to radiotherapy (≥200 mg/m^2^ cumulative dose).
- Hydration with 1500 mL of normal saline solution and 16 mEq magnesium sulfate (MgSO_4_), administered before and following chemotherapy for a total of 3 L of normal saline solution and 32 mEq MgSO_4_, administered intravenously (i.v.) in 4 h.
- Premedication with:
  - Dexamethasone 12 mg i.v.
  - Netupitant 300 mg per OS
  - Omeprazole 40 mg i.v.
  - Mannitol 100 mg i.v.
- The same hydration regimen, magnesium dose, proton pump inhibitors, and corticosteroid administration were repeated on the day after chemotherapy.

### 2.2. Statistical Analyses

Statistical analyses were performed using JASP (version 0.18), with two-sided tests and a significance threshold set at *p* < 0.05. Continuous variables were summarized as median and interquartile range, as most did not meet normality assumptions, which were assessed using the Shapiro–Wilk test and visual inspection of Q–Q plots. Variables with significant skewness underwent natural-log or Box–Cox transformation before inferential testing. Between-group comparisons of normally distributed continuous variables were performed using Student’s two-sample *t*-test, whereas the Wilcoxon rank-sum test was applied to nonparametric data. Categorical variables were compared using the χ^2^ test or, when expected cell counts were <5, Fisher’s exact test.

Multivariate analysis was performed using a logistic regression model, with results expressed as odds ratios and corresponding 95% confidence intervals.

## 3. Results

Table 1a,b describe the demographic, clinical, and laboratory characteristics of the study population. The median age was 64 years (interquartile range [IQR] 57–72), and 27.54% of participants were female. Median height and weight were 170 cm and 73 kg, yielding a BMI of 24.69 kg/m^2^ (IQR 22.13–27.51). Hypertension was the most prevalent comorbidity (29.95%), followed by diabetes (7.73%), while 58.45% of patients were current smokers and 15.94% reported a history of alcohol consumption. The most frequently used medications were RAAS inhibitors (21.26%), proton pump inhibitors (17.39%), and β-blockers (12.56%), with lower frequencies for acetylsalicylic acid (10.63%), diuretics (9.66%), and oral antidiabetic agents (5.8%).

At baseline (cycle 1), median laboratory values included urea 34 mg/dL, hemoglobin 13.9 g/dL, and white blood cell count 7.4 × 10^9^/L, with corresponding neutrophil and lymphocyte counts of 4.8 × 10^9^/L and 1.8 × 10^9^/L. Over subsequent treatment cycles, hemoglobin, leukocyte, neutrophil, and lymphocyte counts progressively declined. At the same time, urea levels fluctuated, and the median estimated glomerular filtration rate (CKD-EPI 2012) decreased modestly from 92.45 mL/min/1.73 m^2^ at cycle 1 to 88.73 mL/min/1.73 m^2^ at cycle 3.

The distribution of CKD stages at baseline according to KDIGO 2024 was reported in Table 1c. Among the 196 patients with available data, 112 (57.14%) were classified as stage 1 (eGFR ≥ 90 mL/min/1.73 m^2^) and 78 (39.80%) as stage 2 (eGFR 60–89 mL/min/1.73 m^2^). Only 3 patients (1.53%) were in stage 3a (eGFR 45–59 mL/min/1.73 m^2^) and 3 (1.53%) in stage 3b (eGFR 30–44 mL/min/1.73 m^2^). Stages 4 and 5 were not observed. Overall, 96.94% of evaluable patients had normal or mildly reduced renal function at baseline, indicating that the subsequent eGFR decline observed across treatment cycles occurred primarily in individuals with preserved kidney function. AKD occurred in 5.3% of patients (11/207; 95% CI 2.7–9.3).

### 3.1. Univariate Analysis of AKD Predictors

The univariate analysis of categorical variables (Table 2a) showed that the use of renin–angiotensin–aldosterone system (RAAS) inhibitors was the only factor significantly associated with the presence of acute kidney disease (AKD) (*p* = 0.04). Patients receiving RAAS inhibitors accounted for 21.3% of the total population, and 5 of these developed AKD. Hypertension demonstrated a borderline association (*p* = 0.07), consistent with its pathophysiological link to chronic kidney injury and the frequent concomitant use of RAAS inhibitors in these patients. All other evaluated categorical variables, including insulin use, proton pump inhibitors, systemic corticosteroids, acetylsalicylic acid, oral antidiabetic agents, alcohol consumption (Potus), opioid analgesics, β-blockers, calcium antagonists, diuretics, diabetes mellitus, current smoking status, and female sex, did not reach statistical significance (*p* ≥ 0.05), indicating no measurable association with AKD in this cohort.

The analysis of continuous variables (Table 2b) revealed a significant association between higher baseline BMI and AKD occurrence (*p* = 0.02), suggesting that overweight and obesity may contribute to increased susceptibility to acute renal injury, potentially through metabolic and hemodynamic mechanisms. Baseline body weight and estimated glomerular filtration rate at cycle 1 (GFR_C1) demonstrated borderline associations with AKD (*p* = 0.06 for both), implying a possible trend toward higher risk in patients with greater body mass or lower baseline kidney function reserve. No significant differences were observed for age (*p* = 0.22) or height (*p* = 0.48), indicating that demographic factors such as chronological age and stature did not influence AKD development in this population.

### 3.2. Multivariate Analysis of AKD Predictors

The multivariate logistic regression model (Table 3) evaluated independent predictors of AKD by including variables with significant or borderline associations in the univariate analysis. None of the examined variables reached statistical significance at the multivariate level. RAASi were not considered although statistically significant in univariate analysis because of collinearity. Baseline BMI demonstrated a non-significant trend toward increased AKD risk (OR 1.36; 95% CI 0.96–1.91; *p* = 0.08), consistent with the univariate findings but attenuated after adjustment for other covariates. Baseline body weight showed no significant association (OR 0.96; 95% CI 0.87–1.05; *p* = 0.34). Baseline serum creatinine at cycle 1 (cycle.1–d0_creatinine) also did not predict AKD occurrence (OR 9.65; 95% CI 0.27–347.27; *p* = 0.22), although the wide confidence interval reflects the small number of AKD events and potential instability of the estimate. The model intercept was significant (*p* < 0.001), indicating a low baseline probability of AKD without the evaluated risk factors. Overall, these results suggest that no variable retained an independent predictive value for AKD after multivariable adjustment, likely due to limited event numbers and collinearity between predictors.

### 3.3. Comparison of eGFR Decline by AKD Status

As shown in Table 4, patients who developed AKD exhibited a greater reduction rate in eGFR over the observation period compared with patients without AKD. In the AKD group, the mean eGFR slope was −0.3963 mL/min/1.73 m^2^ per day (SD 0.2331; median −0.3917; range −0.7435 to −0.0419), indicating a consistent downward trend in renal function across individuals. In contrast, the non-AKD group showed a mean eGFR slope of −0.0672 mL/min/1.73 m^2^ per day (SD 0.2156; median −0.0483; range −0.7130 to 0.5035), with several patients exhibiting stable or even improved eGFR values over time. The Mann–Whitney U test demonstrated a statistically significant difference in eGFR slope distributions between the two groups (U = 1082.0; *p* = 0.0005). These results indicate that AKD was associated with a significantly higher rate of kidney function decline during follow-up, supporting its role as a relevant clinical determinant of short-term deterioration in kidney function.

### 3.4. Comparison of eGFR Decline by CKD Stage

Table 5 reports the distribution of mean eGFR slopes stratified by baseline CKD stage. Among patients with stage 1 CKD, the mean eGFR slope was −0.1119 mL/min/1.73 m^2^ per day (SD 0.1986; median −0.060; range −0.7435 to 0.2832), indicating a mild average decline in kidney function. Stage 2 patients had a mean slope of −0.0592 mL/min/1.73 m^2^ per day (SD 0.2598; median −0.0419; range −0.7130 to 0.5035), suggesting a slower reduction rate than stage 1. In contrast, patients in stage 3a demonstrated a mean positive slope of 0.0853 mL/min/1.73 m^2^ per day (SD 0.1604; median 0.0343; range −0.0435 to 0.2651), while those in stage 3b had a mean slope of 0.0337 mL/min/1.73 m^2^ per day (SD 0.2355; median −0.0849; range −0.1188 to 0.3049).

Overall, baseline CKD stage was not associated with a more rapid decline in eGFR during follow-up. The absence of a progressive negative slope in stages 3a and 3b is likely attributable to the small number of patients in these categories and potential variability in individual measurements, rather than indicating true improvement in renal function.

### 3.5. Acute Kidney Disease Healing Between Treatment Cycles

As shown in Table 6, between cycle 1 (C1) and 2 (C2), 8 patients developed AKD; among them, kidney function recovery was observed in 12.5% of cases, while 87.5% did not revert to baseline renal function within the subsequent cycle. Between C2 and cycle 3 (C3), 3 additional patients developed AKD, and none achieved recovery in the following cycle (0% recovery; 100% non-recovery). These findings indicate that, in this cohort, the majority of AKD episodes persisted beyond the immediate subsequent treatment cycle, and complete recovery from AKD was rare.

It should be noted that the number of available laboratory values differed across timepoints. Missing data reflect the real-world nature of this study, as some tests were not performed at all scheduled cycles due to clinical workflow in an oncologic, not nephrologic, setting. These cases were reported transparently in the tables and were not imputed.

**Figure 1:**
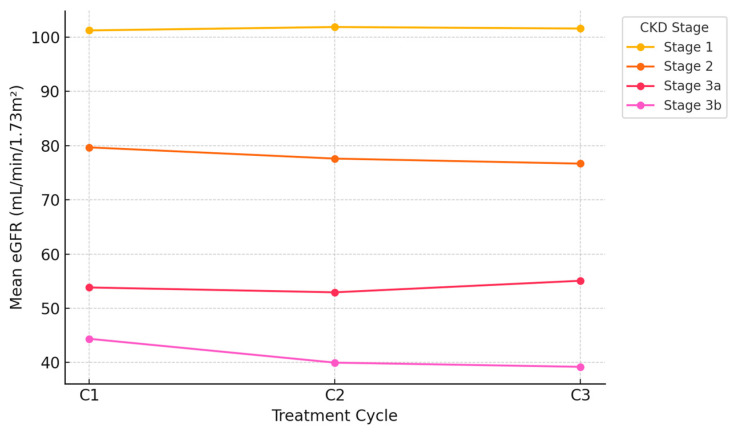
Mean eGFR variation across Treatment Cycles by baseline CKD stage.

Furthermore, the baseline distribution of CKD stages according to the KDIGO classification is reported in Table 7. These findings are also illustrated in Figure 1, which shows the mean eGFR variation across treatment cycles according to baseline CKD stage.

## 4. Discussion

Our study prospectively evaluated the incidence of AKD in patients with LA-HNSCC, receiving high-dose cisplatin–based concurrent CRT, within a standardized hydration protocol that included intravenous magnesium. In a cohort of 207 patients, AKD developed in 11 cases overall; by treatment interval, 8 events occurred between baseline and cycle 1, 3 between cycle 1 and 2, and 0 between cycle 2 and 3. Despite universal magnesium supplementation, recovery at the subsequent cycle was uncommon (9,1%), indicating that, once established, AKD frequently persists beyond the acute window. This pattern is consistent with the ADQI/KDIGO framework in which AKD constitutes a subacute continuum between AKI and CKD, reflecting incomplete tubular repair and maladaptive remodeling rather than transient hemodynamic fluctuation [7,21,26,27].

Univariate analyses identified two variables associated with AKD: use of renin–angiotensin–aldosterone system (RAAS) inhibitors (*p* = 0.04) and higher body-mass index (BMI) (*p* = 0.02). The RAAS signal is mechanistically coherent: efferent arteriolar vasodilation lowers intraglomerular pressure and narrows the kidney’s autoregulatory reserve, blunting the angiotensin II–mediated response to hypovolemia; under CRT conditions, characterized by emesis, mucositis, reduced oral intake, and episodic dehydration, this hemodynamic milieu increases prerenal ischemia and susceptibility of proximal tubular cells to cisplatin injury [28,29,30]. The BMI association is likewise biologically plausible: adiposity is linked to glomerular hyperfiltration and increased single-nephron workload, low-grade systemic inflammation and oxidative stress, tubulointerstitial lipotoxicity, and potentially higher effective drug exposure with body-surface–area dosing, each of which can heighten vulnerability to platinum-induced tubular cytotoxicity [28,29,30]. Borderline associations were observed for body weight and baseline eGFR at cycle 1 (both *p* = 0.06) and for hypertension (*p* = 0.07), aligning with prior evidence that cardiovascular comorbidity and reduced renal reserve modulate cisplatin nephrotoxicity risk [19,28,29,30]. In contrast, insulin, proton-pump inhibitors, systemic steroids, acetylsalicylic acid, diuretics, current smoking, diabetes, and female sex were not associated with AKD in this dataset (all *p* > 0.05). The multivariable model, constrained deliberately because of the low number of events to respect events-per-variable considerations, retained BMI, weight, and baseline creatinine; none achieved statistical significance (BMI OR 1.36, 95% CI 0.96–1.91, *p* = 0.08; weight OR 0.96, 95% CI 0.87–1.05, *p* = 0.34; creatinine OR 9.65, 95% CI 0.27–347.27, *p* = 0.22). The attenuation of univariate signals is most consistent with limited statistical power, wide confidence intervals reflecting imprecision, and collinearity (notably between BMI and weight), rather than with a definitive absence of effect; these features argue for prospective validation in larger cohorts using parsimonious or penalized regression strategies. The eGFR-slope analysis supports clinical relevance. The distribution of slopes differed significantly between patients with and without AKD (Mann–Whitney U = 1082.0; *p* = 0.0005), with the AKD group demonstrating a more negative median slope (−0.3917 vs. −0.0483 mL/min/1.73 m^2^ per day). This quantifies the short-term functional decrement associated with AKD and accords with consensus statements recognizing AKD as prognostically relevant beyond the 7-day AKI window and associated with increased risk of subsequent CKD in a subset of patients [7,21]. By contrast, stratification by baseline CKD stage yielded no significant differences in mean slopes (Kruskal–Wallis H = 3.71; *p* = 0.29), a finding likely influenced by the baseline distribution in this cohort (stage 1, 57.14%; stage 2, 39.80%; stage 3a/3b, ~3% each) and limited power in advanced stages; taken together, the data indicate that AKD status, rather than baseline CKD stage, was more strongly associated with short-term eGFR decline under a magnesium-supplemented hydration protocol. Two additional features distinguish the present series from the others. First, the AKD incidence verified through cycle 2 (5.31% based on baseline→C1 and C1→C2 events) appears lower than rates reported in historical HNC cohorts without uniform magnesium or with heterogeneous hydration strategies, including studies focused on high-dose tri-weekly cisplatin [11,22,31,32,33]. Second, cycle-by-cycle reversibility was low (9.1% recovery at one cycle among verified events), indicating that persistence is the dominant trajectory under real-world CRT conditions even with magnesium support. These observations are coherent with the multi-hit pathophysiology of cisplatin nephrotoxicity, proximal tubular uptake via OCT2/CTR1, mitochondrial dysfunction and reactive oxygen species generation, DNA adduct formation with p53-mediated apoptosis, and activation of inflammatory and pro-fibrotic signaling (TNF-α, IL-6, TGF-β), followed by maladaptive repair and microvascular rarefaction that sustain GFR loss into the AKD interval [9,30,34]. Magnesium prophylaxis is a critical contextual element of our protocol and likely contributed to the relatively low AKD incidence. Mechanistically, magnesium mitigates cisplatin-induced hypomagnesemia (exacerbated by CRT-related gastrointestinal losses) and preserves tubular transport by maintaining TRPM6/epidermal growth factor–dependent magnesium handling in the distal nephron; experimental data indicate that magnesium deficiency increases renal platinum accumulation and down-regulates efflux transporters (MRP2/MRP4/MRP6), whereas repletion restores transporter abundance and attenuates injury [27,35]. Clinically, systematic reviews and large multicenter cohorts show that intravenous magnesium confers additive nephroprotection beyond hydration, with same-day administration associated with approximately 20% lower adjusted odds of moderate-to-severe cisplatin-associated kidney injury or death within 14 days [19,33,36]. This dual mechanism may explain why magnesium prophylaxis appears effective even when standard hydration protocols are applied.

Our findings are consistent with recent large-scale, multicenter evidence supporting a clinically meaningful nephroprotective role of prophylactic IV magnesium in cisplatin-treated patients. In a cohort study including 13,719 adults initiating cisplatin across five US cancer centers, Gupta et al. reported that same-day IV magnesium administration was associated with a 20% lower adjusted odds of developing moderate-to-severe cisplatin-associated AKI or death within 14 days (adjusted OR 0.80; 95% CI 0.66–0.97), after rigorous adjustment for demographics, comorbidities, baseline kidney function, and concurrent nephrotoxic therapies [19]. The benefit was more pronounced in subgroups with preserved baseline eGFR (≥90 mL/min/1.73 m^2^) and higher baseline serum magnesium levels (2.0–2.2 mg/dL), and was evident even in patients receiving standard hydration. These results align with our observation of a comparatively low AKD incidence despite high-dose cisplatin schedules, suggesting that magnesium prophylaxis may mitigate both the acute and subacute phases of platinum nephrotoxicity. Notably, Gupta et al. also highlighted the absence of adverse safety signals and the feasibility of implementation across diverse oncology settings, reinforcing the rationale for systematic inclusion of magnesium in cisplatin hydration protocols [19].

In 2022, Suppadungsuk et al. demonstrated how 16 mEq magnesium added to the saline infusion in preloading regimen was safe and significantly showed a decreased incidence of cis-AKD in patients with head and neck cancer treated with low dose cisplatin chemotherapy, without reducing the longer-term treatment efficacy [37].

A recent study by Okamoto et al. has revealed that premedication with Mg prevented CIN in a dose-dependent manner. In particular in subgroup analysis by forest plot followed by meta-regression analysis, total OR with 95% CI of low Mg dosage administration (<10 mEq) and high Mg dosage administration (≥10 mEq) was 0.35 (0.16–0.77, *p*  =  0.0169) and 0.12 (0.07–0.21, *p*  <  0.0001), respectively, indicating a relationship between Mg dosage and OR (*p*  =  0.0349) [38].

Further studies are required to determine the optimal dose of Mg. In the Gupta et al. multicenter cohort, most centers administered 1–2 g of intravenous magnesium sulfate on the same day as cisplatin, corresponding to approximately 16–32 mEq of elemental magnesium [19]. Still, observations are concordant with this literature: magnesium did not abolish AKD, but the lower incidence relative to the non-magnesium series suggests a real-world risk-reducing effect.

The implications are direct. Surveillance should extend beyond AKI to systematically capture AKD across cycles, because subclinical decrements in GFR, often clinically silent and insidious, may accumulate and convert into CKD, with consequences for cardiovascular risk, quality of life, and eligibility for subsequent oncologic therapies that depend on renal clearance thresholds [7,21]. Patients with elevated BMI, ongoing RAAS blockade, borderline baseline renal reserve, or intense CRT-related toxicities (mucositis, emesis, diarrhea) merit intensified prevention: optimized hydration, continued magnesium, and medication reconciliation to minimize concurrent nephrotoxins (e.g., NSAIDs). RAAS inhibitors can increase AKD risk by reducing efferent arteriolar vasoconstriction, thus lowering intraglomerular pressure when renal perfusion is already compromised by CRT-related hypovolemia and cisplatin toxicity. This effect may be enhanced by concomitant diuretics or targeted agents that impair renal hemodynamics. Observational studies in oncology patients confirm a higher risk of AKI/AKD with RAAS blockade, supporting careful medication review and close renal monitoring during CRT [39,40,41]. Emerging evidence supports novel nephroprotective agents, though these require further validation. Early identification of AKD through sensitive biomarkers such as neutrophil gelatinase-associated lipocalin (NGAL), kidney injury molecule-1 (KIM-1), and interleukin-18 (IL-18) could enable prompt intervention before eGFR decline is apparent and has shown promise for predicting near-term CKD signals after cisplatin. Biomarkers of tubular injury are particularly interesting, as traditional markers like serum creatinine lack sensitivity for early or subclinical kidney injury.

Integrating urinary NGAL, KIM-1, and cell cycle arrest markers (TIMP-2·IGFBP7) into routine surveillance could refine risk prediction and guide individualized adjustments in chemotherapy dosing to balance efficacy with nephrotoxicity risk [42,43].

Our work adds novel insights by (i) reporting AKD incidence with cycle-level recovery/non-recovery under universal magnesium prophylaxis, (ii) coupling binary AKD status with continuous eGFR-slope endpoints to quantify near-term functional impact, and (iii) dissecting univariate vs. multivariable signals in a magnesium-supplemented population.

In addition, our chemotherapy premedication includes mannitol combined with magnesium supplementation and a short scheme (4 or 5 h) of small volume hydration (ranging from 1.9 to 4.3 L), which is known to significantly prevent cisplatin-induced nephrotoxicity [44,45].

**Figure 2:**
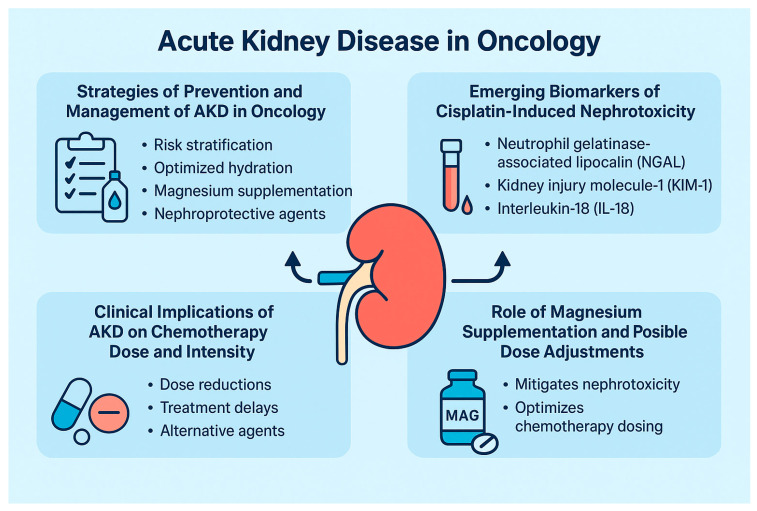
Overview of prevention strategies, biomarkers, and clinical implications of AKD in oncology.

This paper has some limitations. Firstly, the single-center design and the modest sample size with a low absolute number of AKD events reduce the statistical power to identify independent predictors. Furthermore, serum magnesium levels were not systematically measured, precluding a correlation between magnesium status and renal outcomes. Additionally, GFR was only estimated and not measured with exogenous markers, affecting CKD patients with sarcopenia and normal serum creatinine. Lastly, potential unmeasured confounders such as concomitant non-steroidal anti-inflammatory drug (NSAID) use, variability in hydration adherence, and differences in supportive care practices could have influenced renal outcomes. These factors, which reflect the common clinical practice, may partly account for the heterogeneity observed. Future randomized controlled trials should stratify patients by baseline magnesium levels and eGFR to identify subgroups deriving the greatest benefit. An overview of prevention strategies, biomarkers, and clinical implications of AKD in oncology is illustrated in Figure 2.

## 5. Conclusions

In conclusion, in LA-HNSCC patients receiving high-dose cisplatin CRT within a magnesium-supplemented hydration regimen, AKD incidence was lower than in historical non-magnesium cohorts. Still, it remained clinically relevant, with a high persistence rate beyond the immediate subsequent treatment cycle. These findings support the incorporation of magnesium supplementation into standard hydration protocols for cisplatin and highlight the urgent need to implement comprehensive strategies combining early biomarker detection, individualized dosing adjustments, and intensified supportive care to prevent progression of AKD and preserve renal function, thereby safeguarding oncological treatment continuity and long-term patient outcomes.

## Figures and Tables

**Table 1:** (**a**) Descriptive statistics of continuous variables. (**b**) Descriptive analysis of categorical variables. (**c**) Baseline distribution of CKD stages according to KDIGO 2024 classification using the CKD-EPI formula.

**(a)**							
	**Valid**	**Missing**	**Median**	**Minimum**	**Maximum**	**25th** **Percentile**	**75th** **Percentile**
Age	207	0	64.00	28.00	84.00	57.00	72.00
Height	206	1	170.00	150.00	195.00	165.00	178.00
Weight	206	1	73.00	40.00	130.00	62.00	82.00
BMI	206	1	24.69	15.02	40.12	22.13	27.51
Cycle 1 urea	78	129	34.00	14.00	61.00	28.00	39.00
Cycle 1 hb	195	12	13.90	8.50	17.20	12.55	14.80
Cycle 1 wbc	196	11	7.40	2.80	21.80	5.64	9.09
Cycle 1 neut	194	13	4.80	0.60	15.90	3.49	6.27
Cycle 1 lympt	194	13	1.80	0.40	4.50	1.40	2.30
Cycle 2 urea	143	64	33.00	13.00	152.00	24.00	41.00
Cycle 2 hb	190	17	13.00	8.60	17.20	11.80	13.90
Cycle 2 wbc	192	15	4.20	0.80	14.40	3.10	5.40
Cycle 2 neut	193	14	2.60	0.60	28.60	1.80	3.90
Cycle 2 lymph	193	14	0.70	0.10	43.80	0.50	1.00
Cycle 3 urea	104	103	38.50	11.00	142.00	30.00	50.00
Cycle 3 hb	154	53	12.20	3.80	15.80	11.00	13.20
Cycle 3 wbc	157	50	4.05	1.50	28.40	3.10	6.40
Cycle 3 neut	157	50	3.20	0.80	8.65	2.10	4.70
Cycle 3 lymph	157	50	0.50	0.00	18.40	0.30	0.70
Cycle 1 eGFR (CKD-EPI formula 2012)	197	10	92.45	44.37	133.52	84.61	100.09
Cycle 2 eGFR CKD-EPI formula 2012)	193	14	88.78	32.72	136.23	75.03	99.12
Cycle 3 eGFR (CKD-EPI formula 2012)	156	51	88.73	36.38	127.09	74.79	98.76
**(b)**							
**Variable**			**Frequency**			**Percent**	
ASA (Y)			22			10.63	
Oral antidiabetic agents (Y)			12			5.8	
Insulin (Y)			7			3.38	
PPI (Y)			36			17.39	
Steroids (Y)			6			2.9	
Potus (Y)			33			15.94	
Opioid Analgesic(Y)			6			2.9	
RAAS inhibitors (Y)			44			21.26	
Beta blockers (Y)			26			12.56	
Calcium antagonist (Y)			6			2.9	
Diuretics (Y)			20			9.66	
Diabetes (Y)			16			7.73	
Hypertension (Y)			62			29.95	
Smoker (Y)			121			58.45	
Gender (F)			57			27.54	
**(c)**							
**CKD Stage**		**Frequency**	**Percent (%)**	**Valid Percent (%)**		**Cumulative Percent (%)**	
1		112	54.11	57.14		57.14	
2		78	37.68	39.80		96.94	
3a		3	1.45	1.53		98.47	
3b		3	1.45	1.53		100.00	
**Missing**		11	5.31	–		–	
**Total**		207	100.00	–		–	

**Table 2:** (**a**) Univariate analysis of categorical variables by the presence of Acute Kidney Disease (AKD). (**b**) Univariate analysis of continuous variables by the presence of Acute Kidney Disease (AKD).

**(a)**				
**Variable (Yes Category)**	**AKD Yes (n)**	**Total (n)**	**Total (%)**	***p*-Value**
Insulin (Y)	0	7	3.4	0.52
PPI (Y)	1	36	17.4	0.46
Steroids (Y)	0	6	2.9	0.56
ASA (Y)	1	22	10.6	0.87
Oral antidiabetic agents (Y)	1	12	5.8	0.63
Potus (Y)	0	33	15.9	0.14
Opioid Analgesic (Y)	0	6	2.9	0.56
RAAS inhibitors (Y)	5	44	21.3	0.04
Beta blockers (Y)	2	26	12.6	0.56
Calcium antagonist (Y)	0	6	2.9	0.56
Diuretics (Y)	2	20	9.7	0.56
Diabetes (Y)	1	16	7.7	0.86
Hypertension (Y)	6	62	30.0	0.07
Smoker (1)	9	121	58.5	0.26
Gender (F)	2	57	27.5	0.48
**(b)**				
**Variable**	**U**		***p*-Value**	
Age	842.50		0.22	
Height	1208.50		0.48	
Weight	714.50		0.06	
BMI	605.50		**0.02**	
GFR_C1	1368.50		0.06	

**Table 3:** Multivariate logistic regression analysis predicting Acute Kidney Disease (AKD) presence.

Variable	Estimate	SE	Odds Ratio	z	*p*-Value	95% CI (Lower)	95% CI (Upper)
Intercept	−9.60	2.53	6.81 × 10^−5^	−3.79	<0.001	−14.55	−4.64
BMI	0.31	0.18	1.36	1.74	0.08	−0.04	0.65
Weight	−0.04	0.05	0.96	−0.95	0.34	−0.14	0.05
Cycle 1 creatinine	2.27	1.83	9.65	1.24	0.22	−1.32	5.85

**Table 4:** Comparison of mean slopes of eGFR decline (mL/min/1.73 m^2^/day) stratified by presence of AKD (Yes vs. No).

AKD YN	Mean Slope eGFR (mL/min/1.73 m^2^/day)	SD	Min	Median	Max
**No (0)**	−0.0672	0.2156	−0.7130	−0.0483	0.5035
**Yes (1)**	−0.3963	0.2331	−0.7435	−0.3917	−0.0419

The Mann–Whitney U test comparing eGFR slope between AKD groups resulted statistically significant (U statistic = 1082.0; *p*-value = 0.0005).

**Table 5:** Comparison of mean slopes of eGFR decline (mL/min/1.73 m^2^/day) stratified by CKD stages.

CKD Stage	Mean Slope eGFR (mL/min/1.73 m^2^/day)	SD	Min	Median	Max
**1**	−0.1119	0.1986	−0.7435	−0.060	0.2832
**2**	−0.0592	0.2598	−0.7130	−0.0419	0.5035
**3a**	0.0853	0.1604	−0.0435	0.0343	0.2651
**3b**	0.0337	0.2355	−0.1188	−0.0849	0.3049

Kruskal–Wallis test comparing eGFR slopes across CKD stages was not statistically significant (H statistic = 3.71; *p*-value = 0.29).

**Table 6:** Acute Kidney Disease healing Between Treatment Cycles.

Interval	Patients with AKD	%Recover	%Not Recover
C1→C2	8	12.5	87.5
C2→C3	3	0.0	100.0

The table presents the number and percentage of patients who developed AKD between treatment cycles C1→C2 and C2→C3.

**Table 7:** Baseline distribution of CKD stages according to KDIGO classification.

CKD Stage	Frequency	Percent (%)	Valid Percent (%)	Cumulative Percent (%)
1	112	54.11	57.14	57.14
2	78	37.68	39.80	96.94
3a	3	1.45	1.53	98.47
3b	3	1.45	1.53	100.00
**Missing**	11	5.31	–	–
**Total**	207	100.00	–	–

## Data Availability

The datasets generated and analyzed during the current study are not publicly available due to patient privacy and ethical restrictions, but are available from the corresponding author on reasonable request.
